# 5,5-Bis(4-methoxy­phen­yl)-2,8-bis­[3-(trifluoro­meth­yl)phen­yl]-5*H*-cyclo­penta­[2,1*-b*:3,4*-b*’]dipyridine

**DOI:** 10.1107/S160053680803420X

**Published:** 2008-10-25

**Authors:** Katsuhiko Ono, Masaaki Tomura, Katsuhiro Saito

**Affiliations:** a Department of Materials Science and Engineering, Nagoya Institute of Technology, Gokiso, Showa-ku, Nagoya 466-8555, Japan; b Institute for Molecular Science, Myodaiji, Okazaki 444-8585, Japan

## Abstract

The title compound, C_39_H_26_F_6_N_2_O_2_, showed two melting transitions 477.4 and 506.5 K in a differential scanning calorimetry (DSC) study. The first of these can be attributed to a melting phase transition arising from the rotation of two trifluoro­methyl groups. In the crystal structure, both trifluoro­methyl groups are disordered over two sites with occupancy factors of 0.660 (17) and 0.340 (17) for the major and minor orientations, respectively. The introduction of trifluoro­methyl groups inhibits π-stacking between the diaza­fluorene (cyclo­penta­[2,1*-b*:3,4*-b*’]dipyridine) units. Three short F⋯O contacts between 2.80 (3) and 2.95 (1) Å are observed in the crystal structure.

## Related literature

The synthesis and thermal properties of the title compound were reported by Ono *et al.* (2007 ▶). For related literature on mol­ecular and crystal structures, including the 4,5-diaza­fluorene system, see: Ono & Saito (2008 ▶).
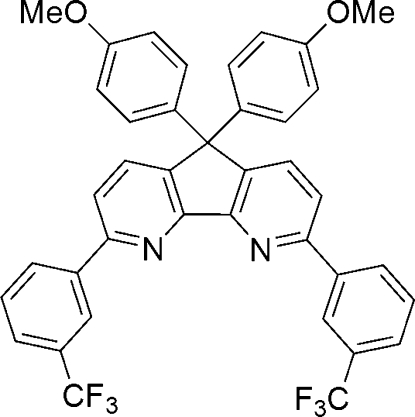


## Experimental

### 

#### Crystal data

C_39_H_26_F_6_N_2_O_2_
*M* *_r_* = 668.62
Orthorhombic, 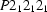

*a* = 9.283 (2) Å
*b* = 12.357 (3) Å
*c* = 26.941 (6) Å
*V* = 3090.4 (12) Å^3^
*Z* = 4
Mo *K*α radiation
μ = 0.11 mm^−1^
*T* = 173 (1) K
0.38 × 0.30 × 0.20 mm

#### Data collection

Rigaku/MSC Mercury CCD diffractometer
Absorption correction: none
20599 measured reflections
3171 independent reflections
2849 reflections with *I* > 2σ(*I*)
*R*_int_ = 0.040

#### Refinement

*R*[*F* ^2^ > 2σ(*F* ^2^)] = 0.056
*wR*(*F* ^2^) = 0.146
*S* = 1.05
3171 reflections
498 parameters
24 restraints
H-atom parameters constrained
Δρ_max_ = 0.28 e Å^−3^
Δρ_min_ = −0.25 e Å^−3^

### 

Data collection: *CrystalClear* (Rigaku/MSC, 2001 ▶); cell refinement: *CrystalClear*; data reduction: *CrystalClear*; program(s) used to solve structure: *SHELXS97* (Sheldrick, 2008 ▶); program(s) used to refine structure: *SHELXL97* (Sheldrick, 2008 ▶); molecular graphics: *PLATON* (Spek, 2003 ▶) and *Mercury* (Macrae *et al.*, 2006 ▶); software used to prepare material for publication: *WinGX* (Farrugia, 1999 ▶).

## Supplementary Material

Crystal structure: contains datablocks I, global. DOI: 10.1107/S160053680803420X/hb2809sup1.cif

Structure factors: contains datablocks I. DOI: 10.1107/S160053680803420X/hb2809Isup2.hkl

Additional supplementary materials:  crystallographic information; 3D view; checkCIF report

## Figures and Tables

**Fig. 1.**
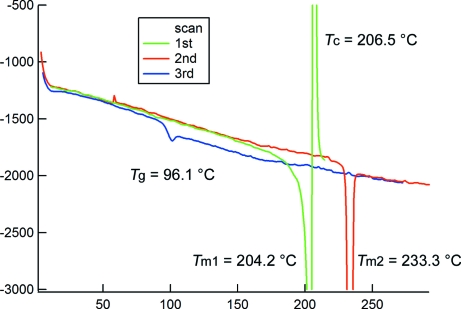
The DSC profile of (I).

**Fig. 2.**
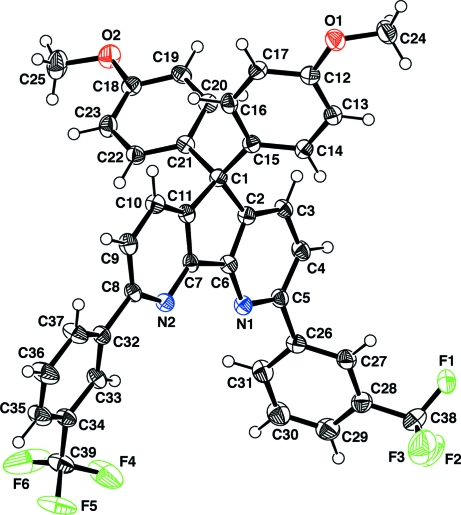
The molecular structure of (I), with 50% probability displacement elipsoids for non-H atoms and H atoms are shown as small spheres of arbitrary radius. The disordered fluorine atoms (F7–F12) of the two trifluoromethyl groups are omitted for clarity.

**Fig. 3.**
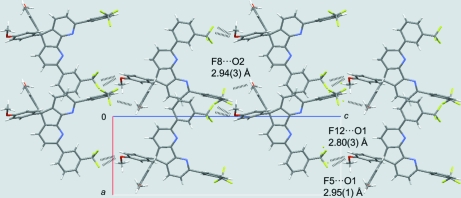
The packing diagram of (I).

## supplementary crystallographic information

### Comment

The title compound, (I), was prepared as a new electron-transporting material in the study of organic electroluminescent (EL) devices (Ono *et al.*, 2007).  A differential scanning calorimetry (DSC) study of (I) revealed novel thermal behavior (Fig. 1). The first heating profile showed a first melting point of 477.4 K and a crystallization temperature of 479.7 K. After cooling, the second heating profile exhibited a second melting point of 506.5 K.  Furthermore, the third heating profile displayed only a glass transition temperature of 369.3 K. The first melting point can be attributed to a phase transition arising from rotation of two trifluoromethyl groups.

The molecular structure of (I) is shown in Fig. 2. The 4,5-diazafluorene (cyclopenta[2,1*-b*:3,4*-b*']dipyridine) ring system is almost planar with an r.m.s. deviation of 0.052Å.  The dihedral angles between the 4,5-diazafluorene ring system and the pendant trifluoromethylphenyl groups are 19.5 (1) ° and 62.3 (1) °. Both the trifluoromethyl groups are disordered over two sites with occupancy factors of 0.660 (17):0.340 (17). The crystal structure is characterized by an arrangement of the molecules along the *c* axis (Fig. 3). In the molecular arrangement, significant π–π overlapping between the 4,5-diazafluorene moieties is not found, although it was observed in the crystal of a 2,8-diphenyl derivative (Ono & Saito, 2008). Three short F···O contacts between 2.80 (3) and 2.95 (1) Å are observed in the crystal of (I).

### Experimental

The title compound (I) was synthesized by the direct arylation reaction of mono-substituted compound (II), which was also prepared by the direct arylation of 5,5-bis(4-methoxyphenyl)-5*H*-cyclopenta[2,1*-b*:3,4*-b*']dipyridine (III). The direct arylation of compound (II) was performed as follows: *n*BuLi in hexane (1.60 *M*, 3.3 ml, 5.2 mmol) was added dropwise to a solution of 3-bromobenzotrifluoride (0.66 ml, 4.8 mmol) in dry ether (20 ml) at 195 K under nitrogen. The solution was stirred at 195 K for 30 min and at 0 °C for 30 min. Compound (II) (1.52 g, 4.0 mmol) and dry toluene (30 ml) were added to the solution at 253 K. The reaction mixture was stirred at 253 K for 30 min and at room temperature overnight. The mixture was poured into water and dichloromethane was added. The organic layer was separated and aqueous layer was extracted with dichloromethane (× 2). Manganese(IV) oxide (MnO_2_) (10 g) was added to the combined organic solution and the mixture was stirred for 30 min. Further, anhydrous magnesium sulfate (MgSO_4_) (10 g) was added and the mixture was stirred for 30 min. After filtration and condensation, the residue was chromatographed on silica gel (hexane/ethyl acetate = 3:1 v/v) to afford the title compound as a white solid (1.28 g, 39%). Colorless prisms of (I) suitable for X-ray analysis were grown from an acetonitrile solution.

### Refinement

Anomalous dispersion was negligible and Friedel pairs were merged before refinement.

All the H atoms were placed in geometrically calculated positions, with C—H = 0.95 (phenyl) and 0.98 (methyl) Å, and refined using a riding model with *U*_iso_(H) = 1.2*U*_eq_(C) (phenyl) and 1.5*U*_eq_(C) (methyl).

### Crystal data

**Table:**

C_39_H_26_F_6_N_2_O_2_	*D*_x_ = 1.437 Mg m^−^^3^
*M**_r_* = 668.62	Melting point: 483 K
Orthorhombic, *P*2_1_2_1_2_1_	Mo *K*α radiation, λ = 0.71073 Å
Hall symbol: P 2ac 2ab	Cell parameters from 9363 reflections
*a* = 9.283 (2) Å	θ = 3.0–27.5°
*b* = 12.357 (3) Å	µ = 0.11 mm^−^^1^
*c* = 26.941 (6) Å	*T* = 173 K
*V* = 3090.4 (12) Å^3^	Prism, colorless
*Z* = 4	0.38 × 0.30 × 0.20 mm
*F*(000) = 1376	

### Data collection

**Table:**

Rigaku/MSC Mercury CCD diffractometer	2849 reflections with *I* > 2σ(*I*)
Radiation source: Rotating Anode	*R*_int_ = 0.040
Graphite Monochromator	θ_max_ = 27.5°, θ_min_ = 3.0°
Detector resolution: 14.7059 pixels mm^-1^	*h* = −11→11
φ and ω scans	*k* = −15→13
20599 measured reflections	*l* = −30→34
3171 independent reflections	

### Refinement

**Table:**

Refinement on *F*^2^	Secondary atom site location: difference Fourier map
Least-squares matrix: full	Hydrogen site location: inferred from neighbouring sites
*R*[*F*^2^ > 2σ(*F*^2^)] = 0.056	H-atom parameters constrained
*wR*(*F*^2^) = 0.146	*w* = 1/[σ^2^(*F*_o_^2^) + (0.1005*P*)^2^ + 0.7722*P*] where *P* = (*F*_o_^2^ + 2*F*_c_^2^)/3
*S* = 1.05	(Δ/σ)_max_ = 0.004
3171 reflections	Δρ_max_ = 0.28 e Å^−^^3^
498 parameters	Δρ_min_ = −0.25 e Å^−^^3^
24 restraints	Extinction correction: SHELXL97 (Sheldrick, 2008), Fc^*^=kFc[1+0.001xFc^2^λ^3^/sin(2θ)]^-1/4^
Primary atom site location: structure-invariant direct methods	Extinction coefficient: 0.0027 (15)

### Special details

**Table:**

Experimental. IR (KBr, cm^-1^): 1607, 1574, 1510, 1337, 1298, 1256, 1175, 1130, 1073, 1030, 837, 808, 700; ^1^H NMR (CDCl_3_, δ p.p.m): 3.78 (s, 6H), 6.81 (d, 4H, J = 8.8 Hz), 7.17 (d, 4H, J = 8.8 Hz), 7.62–7.73 (m, 4H), 7.79 (d, 2H, J = 8.1 Hz), 7.88 (d, 2H, J = 8.1 Hz), 8.45 (m, 4H). ^13^C NMR (CDCl_3_, δ p.p.m): 55.3, 59.9, 114.1, 121.1, 124.2 (q, J = 3.7 Hz), 124.2 (q, J = 272.5 Hz), 125.8 (q, J = 3.6 Hz), 128.9, 129.2, 130.8, 131.1 (q, J = 30.5 Hz), 134.6, 135.4, 139.7, 146.3, 156.6, 157.4, 158.9; MS (EI): m/*z* 668 (*M*^+^, 100). Anal. Calcd for C_39_H_26_F_6_N_2_O_2_: C, 70.06; H, 3.92; N, 4.19. Found: C, 70.32; H, 3.81; N, 4.24.
Geometry. All e.s.d.'s (except the e.s.d. in the dihedral angle between two l.s. planes) are estimated using the full covariance matrix. The cell e.s.d.'s are taken into account individually in the estimation of e.s.d.'s in distances, angles and torsion angles; correlations between e.s.d.'s in cell parameters are only used when they are defined by crystal symmetry. An approximate (isotropic) treatment of cell e.s.d.'s is used for estimating e.s.d.'s involving l.s. planes.
Refinement. Refinement of *F*^2^ against ALL reflections. The weighted *R*-factor *wR* and goodness of fit *S* are based on *F*^2^, conventional *R*-factors *R* are based on *F*, with *F* set to zero for negative *F*^2^. The threshold expression of *F*^2^ > σ(*F*^2^) is used only for calculating *R*-factors(gt) *etc*. and is not relevant to the choice of reflections for refinement. *R*-factors based on *F*^2^ are statistically about twice as large as those based on *F*, and *R*- factors based on ALL data will be even larger. The fluorine atoms of the two trifluoromethyl groups are disordered over two sites (F1–F6 and F7–F12) with occupancies of 0.66 (2):0.34 (2). The values were determined by refining site occupancies.

### Fractional atomic coordinates and isotropic or equivalent isotropic displacement parameters (Å^2^)

**Table:**

	*x*	*y*	*z*	*U*_iso_*/*U*_eq_	Occ. (<1)
C1	−0.0417 (4)	−0.0056 (3)	0.68651 (14)	0.0225 (8)	
C2	−0.1083 (4)	−0.0379 (3)	0.73646 (14)	0.0221 (8)	
C3	−0.2401 (4)	−0.0855 (3)	0.74784 (15)	0.0243 (8)	
H3	−0.3053	−0.1065	0.7224	0.029*	
C4	−0.2729 (5)	−0.1013 (3)	0.79725 (15)	0.0274 (9)	
H4	−0.3601	−0.1365	0.8061	0.033*	
C5	−0.1782 (4)	−0.0656 (3)	0.83462 (14)	0.0241 (8)	
C6	−0.0207 (4)	−0.0069 (3)	0.77591 (14)	0.0217 (8)	
C7	0.1139 (4)	0.0386 (3)	0.75513 (14)	0.0211 (8)	
C8	0.3389 (4)	0.1127 (3)	0.75455 (15)	0.0227 (8)	
C9	0.3418 (5)	0.1070 (3)	0.70272 (16)	0.0284 (9)	
H9	0.4251	0.1307	0.6853	0.034*	
C10	0.2246 (5)	0.0673 (3)	0.67642 (15)	0.0264 (8)	
H10	0.2256	0.0628	0.6412	0.032*	
C11	0.1057 (4)	0.0345 (3)	0.70387 (14)	0.0219 (8)	
C12	−0.0196 (5)	−0.2558 (3)	0.57401 (15)	0.0251 (8)	
C13	−0.0576 (4)	−0.2838 (3)	0.62200 (15)	0.0247 (8)	
H13	−0.0803	−0.3568	0.6298	0.030*	
C14	−0.0625 (4)	−0.2046 (3)	0.65879 (15)	0.0237 (8)	
H14	−0.0888	−0.2247	0.6916	0.028*	
C15	−0.0298 (4)	−0.0961 (3)	0.64873 (14)	0.0229 (8)	
C16	0.0115 (4)	−0.0708 (3)	0.59949 (15)	0.0258 (9)	
H16	0.0345	0.0019	0.5912	0.031*	
C17	0.0190 (5)	−0.1496 (3)	0.56326 (15)	0.0266 (9)	
H17	0.0507	−0.1312	0.5308	0.032*	
C18	−0.3207 (4)	0.2477 (3)	0.62886 (15)	0.0265 (9)	
C19	−0.3525 (4)	0.1388 (3)	0.62095 (15)	0.0262 (9)	
H19	−0.4372	0.1190	0.6034	0.031*	
C20	−0.2610 (4)	0.0602 (3)	0.63860 (15)	0.0256 (9)	
H20	−0.2836	−0.0138	0.6331	0.031*	
C21	−0.1352 (4)	0.0870 (3)	0.66452 (14)	0.0215 (8)	
C22	−0.1038 (5)	0.1955 (3)	0.67151 (15)	0.0264 (9)	
H22	−0.0184	0.2153	0.6887	0.032*	
C23	−0.1952 (5)	0.2765 (3)	0.65377 (16)	0.0296 (9)	
H23	−0.1719	0.3506	0.6587	0.036*	
C24	−0.0996 (6)	−0.4215 (4)	0.53919 (18)	0.0383 (11)	
H24A	−0.0856	−0.4665	0.5096	0.058*	
H24B	−0.2013	−0.4010	0.5418	0.058*	
H24C	−0.0712	−0.4624	0.5688	0.058*	
C25	−0.3891 (6)	0.4316 (3)	0.6160 (2)	0.0477 (13)	
H25A	−0.4691	0.4739	0.6022	0.072*	
H25B	−0.3007	0.4481	0.5976	0.072*	
H25C	−0.3760	0.4501	0.6510	0.072*	
C26	−0.2127 (4)	−0.0782 (3)	0.88795 (14)	0.0250 (8)	
C27	−0.2372 (5)	−0.1807 (4)	0.90810 (15)	0.0302 (9)	
H27	−0.2328	−0.2428	0.8874	0.036*	
C28	−0.2681 (5)	−0.1925 (4)	0.95836 (16)	0.0336 (10)	
C29	−0.2793 (5)	−0.1024 (4)	0.98864 (17)	0.0384 (11)	
H29	−0.3024	−0.1104	1.0228	0.046*	
C30	−0.2569 (5)	−0.0014 (4)	0.96899 (17)	0.0395 (11)	
H30	−0.2646	0.0606	0.9897	0.047*	
C31	−0.2231 (5)	0.0109 (4)	0.91903 (16)	0.0325 (10)	
H31	−0.2070	0.0813	0.9060	0.039*	
C32	0.4591 (4)	0.1623 (3)	0.78298 (14)	0.0237 (8)	
C33	0.4380 (4)	0.1942 (3)	0.83220 (16)	0.0270 (9)	
H33	0.3477	0.1814	0.8477	0.032*	
C34	0.5474 (5)	0.2441 (3)	0.85863 (15)	0.0274 (9)	
C35	0.6800 (5)	0.2636 (4)	0.83707 (17)	0.0366 (11)	
H35	0.7541	0.2990	0.8552	0.044*	
C36	0.7027 (5)	0.2308 (4)	0.78883 (18)	0.0446 (13)	
H36	0.7941	0.2424	0.7739	0.053*	
C37	0.5943 (5)	0.1811 (4)	0.76177 (17)	0.0346 (10)	
H37	0.6120	0.1595	0.7285	0.041*	
C38	−0.2852 (7)	−0.3031 (4)	0.97895 (19)	0.0466 (12)	
C39	0.5217 (5)	0.2783 (4)	0.91103 (17)	0.0375 (10)	
N1	−0.0507 (4)	−0.0179 (3)	0.82383 (12)	0.0236 (7)	
N2	0.2259 (4)	0.0768 (3)	0.78144 (12)	0.0233 (7)	
O1	−0.0136 (4)	−0.3268 (2)	0.53532 (11)	0.0343 (7)	
O2	−0.4209 (3)	0.3188 (2)	0.61156 (12)	0.0369 (8)	
F1	−0.3382 (15)	−0.3725 (10)	0.9461 (4)	0.071 (3)	0.660 (17)
F2	−0.3476 (14)	−0.3106 (8)	1.0210 (3)	0.075 (3)	0.660 (17)
F3	−0.1500 (9)	−0.3518 (9)	0.9890 (5)	0.075 (3)	0.660 (17)
F4	0.4349 (13)	0.2060 (11)	0.9350 (2)	0.090 (4)	0.660 (17)
F5	0.6399 (7)	0.2656 (8)	0.9387 (4)	0.056 (2)	0.660 (17)
F6	0.4708 (16)	0.3715 (9)	0.9164 (3)	0.107 (5)	0.660 (17)
F7	−0.422 (3)	−0.3074 (18)	1.0018 (14)	0.142 (12)	0.340 (17)
F8	−0.198 (4)	−0.3251 (18)	1.0103 (10)	0.116 (10)	0.340 (17)
F9	−0.307 (3)	−0.383 (2)	0.9498 (6)	0.074 (7)	0.340 (17)
F10	0.559 (3)	0.3875 (16)	0.9153 (8)	0.124 (9)	0.340 (17)
F11	0.3896 (12)	0.2861 (18)	0.9235 (6)	0.069 (6)	0.340 (17)
F12	0.588 (4)	0.238 (3)	0.9431 (10)	0.155 (13)	0.340 (17)

### Atomic displacement parameters (Å^2^)

**Table:**

	*U*^11^	*U*^22^	*U*^33^	*U*^12^	*U*^13^	*U*^23^
C1	0.0176 (19)	0.0241 (19)	0.0257 (19)	−0.0006 (15)	−0.0014 (16)	0.0025 (15)
C2	0.0169 (19)	0.0242 (19)	0.0252 (19)	−0.0001 (15)	0.0009 (16)	0.0004 (14)
C3	0.020 (2)	0.026 (2)	0.0266 (19)	−0.0040 (15)	−0.0008 (16)	−0.0027 (15)
C4	0.023 (2)	0.029 (2)	0.031 (2)	−0.0062 (17)	0.0011 (18)	−0.0025 (16)
C5	0.023 (2)	0.0240 (19)	0.025 (2)	−0.0035 (15)	0.0013 (17)	−0.0006 (14)
C6	0.0180 (19)	0.0228 (19)	0.0243 (19)	0.0001 (14)	0.0018 (16)	−0.0020 (14)
C7	0.0155 (19)	0.0203 (18)	0.028 (2)	−0.0030 (14)	−0.0008 (16)	−0.0001 (14)
C8	0.016 (2)	0.024 (2)	0.028 (2)	−0.0032 (15)	−0.0003 (16)	−0.0022 (15)
C9	0.018 (2)	0.032 (2)	0.035 (2)	−0.0064 (16)	0.0030 (18)	0.0009 (17)
C10	0.024 (2)	0.030 (2)	0.0254 (19)	−0.0004 (16)	0.0033 (17)	−0.0005 (16)
C11	0.0164 (19)	0.0226 (19)	0.027 (2)	−0.0007 (14)	0.0007 (16)	−0.0011 (15)
C12	0.021 (2)	0.028 (2)	0.027 (2)	0.0017 (16)	−0.0005 (17)	−0.0002 (15)
C13	0.023 (2)	0.0229 (19)	0.028 (2)	0.0017 (15)	0.0007 (17)	−0.0014 (15)
C14	0.017 (2)	0.027 (2)	0.028 (2)	−0.0008 (15)	0.0004 (16)	0.0043 (15)
C15	0.019 (2)	0.0254 (19)	0.0242 (19)	−0.0010 (15)	−0.0004 (16)	0.0004 (14)
C16	0.022 (2)	0.0231 (19)	0.032 (2)	−0.0031 (16)	0.0001 (17)	0.0008 (15)
C17	0.029 (2)	0.028 (2)	0.024 (2)	−0.0024 (17)	−0.0002 (18)	0.0028 (15)
C18	0.022 (2)	0.029 (2)	0.028 (2)	0.0028 (16)	−0.0010 (17)	0.0028 (16)
C19	0.018 (2)	0.031 (2)	0.029 (2)	−0.0016 (16)	−0.0050 (17)	−0.0021 (16)
C20	0.024 (2)	0.027 (2)	0.0256 (19)	0.0003 (16)	0.0009 (17)	−0.0011 (15)
C21	0.019 (2)	0.0231 (19)	0.0228 (19)	0.0021 (15)	0.0016 (15)	−0.0022 (14)
C22	0.023 (2)	0.026 (2)	0.030 (2)	−0.0011 (16)	−0.0051 (17)	0.0005 (16)
C23	0.025 (2)	0.026 (2)	0.038 (2)	−0.0023 (16)	0.0002 (19)	0.0042 (17)
C24	0.044 (3)	0.031 (2)	0.040 (2)	−0.007 (2)	0.000 (2)	−0.0066 (19)
C25	0.038 (3)	0.029 (2)	0.076 (4)	0.004 (2)	−0.011 (3)	0.013 (2)
C26	0.0163 (19)	0.034 (2)	0.025 (2)	−0.0038 (16)	0.0030 (16)	−0.0009 (16)
C27	0.029 (2)	0.034 (2)	0.028 (2)	−0.0080 (17)	−0.0002 (18)	0.0006 (16)
C28	0.031 (2)	0.039 (2)	0.031 (2)	−0.0077 (19)	0.000 (2)	0.0056 (18)
C29	0.038 (3)	0.048 (3)	0.029 (2)	−0.007 (2)	0.006 (2)	−0.0015 (19)
C30	0.041 (3)	0.041 (3)	0.036 (2)	−0.005 (2)	0.007 (2)	−0.007 (2)
C31	0.033 (2)	0.033 (2)	0.032 (2)	−0.0027 (19)	0.0080 (19)	−0.0064 (18)
C32	0.018 (2)	0.026 (2)	0.027 (2)	−0.0035 (15)	−0.0008 (16)	−0.0011 (15)
C33	0.016 (2)	0.030 (2)	0.034 (2)	−0.0025 (15)	−0.0013 (17)	−0.0039 (16)
C34	0.021 (2)	0.033 (2)	0.027 (2)	−0.0036 (17)	−0.0029 (17)	−0.0030 (16)
C35	0.023 (2)	0.052 (3)	0.035 (2)	−0.013 (2)	−0.0026 (19)	−0.004 (2)
C36	0.024 (2)	0.073 (4)	0.036 (3)	−0.020 (2)	0.002 (2)	−0.010 (2)
C37	0.023 (2)	0.051 (3)	0.029 (2)	−0.0058 (19)	0.0029 (18)	−0.0136 (19)
C38	0.060 (3)	0.046 (3)	0.033 (2)	−0.008 (3)	−0.003 (2)	0.013 (2)
C39	0.026 (2)	0.055 (3)	0.031 (2)	−0.004 (2)	−0.0031 (19)	−0.010 (2)
N1	0.0190 (17)	0.0266 (17)	0.0253 (17)	−0.0033 (13)	0.0040 (14)	−0.0002 (13)
N2	0.0191 (17)	0.0255 (17)	0.0251 (16)	−0.0004 (13)	0.0001 (14)	−0.0011 (12)
O1	0.043 (2)	0.0291 (16)	0.0307 (15)	−0.0046 (13)	0.0021 (14)	−0.0048 (12)
O2	0.0261 (17)	0.0336 (17)	0.051 (2)	0.0062 (13)	−0.0094 (15)	0.0072 (14)
F1	0.103 (6)	0.037 (4)	0.072 (5)	−0.032 (4)	−0.048 (4)	0.020 (3)
F2	0.122 (8)	0.061 (4)	0.041 (3)	−0.009 (6)	0.028 (4)	0.014 (3)
F3	0.065 (4)	0.054 (5)	0.107 (8)	0.009 (3)	−0.020 (4)	0.021 (5)
F4	0.100 (7)	0.139 (8)	0.031 (3)	−0.077 (7)	0.024 (4)	−0.021 (4)
F5	0.025 (3)	0.114 (5)	0.030 (3)	0.006 (3)	−0.007 (2)	−0.026 (3)
F6	0.190 (13)	0.088 (6)	0.042 (4)	0.105 (7)	−0.025 (6)	−0.020 (4)
F7	0.137 (16)	0.061 (9)	0.23 (3)	0.015 (13)	0.144 (18)	0.045 (15)
F8	0.18 (2)	0.060 (12)	0.108 (19)	−0.014 (15)	−0.081 (18)	0.035 (11)
F9	0.16 (2)	0.050 (8)	0.016 (5)	0.015 (9)	0.021 (8)	0.016 (4)
F10	0.17 (2)	0.097 (10)	0.102 (12)	−0.089 (13)	0.058 (12)	−0.076 (9)
F11	0.031 (5)	0.114 (15)	0.061 (8)	−0.013 (6)	0.023 (5)	−0.051 (9)
F12	0.18 (2)	0.22 (2)	0.057 (10)	0.17 (2)	−0.014 (16)	−0.015 (15)

### Geometric parameters (Å, °)

**Table:**

C1—C15	1.516 (5)	C24—O1	1.421 (5)
C1—C11	1.529 (5)	C24—H24A	0.9800
C1—C2	1.534 (5)	C24—H24B	0.9800
C1—C21	1.553 (5)	C24—H24C	0.9800
C2—C3	1.391 (5)	C25—O2	1.430 (6)
C2—C6	1.392 (6)	C25—H25A	0.9800
C3—C4	1.379 (6)	C25—H25B	0.9800
C3—H3	0.9500	C25—H25C	0.9800
C4—C5	1.407 (6)	C26—C31	1.387 (6)
C4—H4	0.9500	C26—C27	1.396 (6)
C5—N1	1.354 (5)	C27—C28	1.392 (6)
C5—C26	1.480 (5)	C27—H27	0.9500
C6—N1	1.328 (5)	C28—C29	1.385 (6)
C6—C7	1.480 (5)	C28—C38	1.483 (7)
C7—N2	1.344 (5)	C29—C30	1.371 (7)
C7—C11	1.384 (5)	C29—H29	0.9500
C8—N2	1.350 (5)	C30—C31	1.390 (7)
C8—C9	1.398 (6)	C30—H30	0.9500
C8—C32	1.486 (5)	C31—H31	0.9500
C9—C10	1.388 (6)	C32—C37	1.398 (6)
C9—H9	0.9500	C32—C33	1.397 (6)
C10—C11	1.389 (6)	C33—C34	1.385 (6)
C10—H10	0.9500	C33—H33	0.9500
C12—O1	1.364 (5)	C34—C35	1.383 (6)
C12—C13	1.384 (6)	C34—C39	1.493 (6)
C12—C17	1.391 (5)	C35—C36	1.378 (7)
C13—C14	1.393 (6)	C35—H35	0.9500
C13—H13	0.9500	C36—C37	1.387 (6)
C14—C15	1.401 (5)	C36—H36	0.9500
C14—H14	0.9500	C37—H37	0.9500
C15—C16	1.416 (6)	C38—F8	1.20 (2)
C16—C17	1.380 (5)	C38—F9	1.28 (2)
C16—H16	0.9500	C38—F2	1.275 (10)
C17—H17	0.9500	C38—F1	1.328 (11)
C18—O2	1.362 (5)	C38—F3	1.418 (10)
C18—C23	1.391 (6)	C38—F7	1.417 (19)
C18—C19	1.393 (6)	C39—F12	1.17 (2)
C19—C20	1.376 (6)	C39—F11	1.275 (11)
C19—H19	0.9500	C39—F6	1.253 (8)
C20—C21	1.401 (6)	C39—F5	1.336 (9)
C20—H20	0.9500	C39—F10	1.399 (16)
C21—C22	1.385 (5)	C39—F4	1.366 (8)
C22—C23	1.396 (6)	F5—O1^i^	2.953 (11)
C22—H22	0.9500	F8—O2^ii^	2.94 (3)
C23—H23	0.9500	F12—O1^i^	2.80 (3)
			
C15—C1—C11	112.3 (3)	C18—C23—H23	120.3
C15—C1—C2	115.2 (3)	C22—C23—H23	120.3
C11—C1—C2	100.2 (3)	O1—C24—H24A	109.5
C15—C1—C21	109.2 (3)	O1—C24—H24B	109.5
C11—C1—C21	112.2 (3)	H24A—C24—H24B	109.5
C2—C1—C21	107.5 (3)	O1—C24—H24C	109.5
C3—C2—C6	117.5 (4)	H24A—C24—H24C	109.5
C3—C2—C1	131.1 (4)	H24B—C24—H24C	109.5
C6—C2—C1	111.3 (3)	O2—C25—H25A	109.5
C4—C3—C2	117.8 (4)	O2—C25—H25B	109.5
C4—C3—H3	121.1	H25A—C25—H25B	109.5
C2—C3—H3	121.1	O2—C25—H25C	109.5
C3—C4—C5	120.6 (4)	H25A—C25—H25C	109.5
C3—C4—H4	119.7	H25B—C25—H25C	109.5
C5—C4—H4	119.7	C31—C26—C27	118.3 (4)
N1—C5—C4	121.9 (4)	C31—C26—C5	121.1 (4)
N1—C5—C26	116.3 (3)	C27—C26—C5	120.6 (4)
C4—C5—C26	121.7 (4)	C28—C27—C26	120.5 (4)
N1—C6—C2	126.3 (4)	C28—C27—H27	119.8
N1—C6—C7	125.7 (3)	C26—C27—H27	119.8
C2—C6—C7	108.0 (3)	C29—C28—C27	120.3 (4)
N2—C7—C11	125.6 (4)	C29—C28—C38	120.9 (4)
N2—C7—C6	125.9 (3)	C27—C28—C38	118.8 (4)
C11—C7—C6	108.5 (3)	C30—C29—C28	119.6 (4)
N2—C8—C9	122.3 (4)	C30—C29—H29	120.2
N2—C8—C32	116.3 (3)	C28—C29—H29	120.2
C9—C8—C32	121.4 (4)	C29—C30—C31	120.5 (4)
C10—C9—C8	120.8 (4)	C29—C30—H30	119.8
C10—C9—H9	119.6	C31—C30—H30	119.8
C8—C9—H9	119.6	C26—C31—C30	120.9 (4)
C11—C10—C9	117.0 (4)	C26—C31—H31	119.6
C11—C10—H10	121.5	C30—C31—H31	119.6
C9—C10—H10	121.5	C37—C32—C33	117.8 (4)
C7—C11—C10	118.5 (4)	C37—C32—C8	122.1 (4)
C7—C11—C1	111.5 (3)	C33—C32—C8	120.0 (4)
C10—C11—C1	130.0 (4)	C34—C33—C32	120.7 (4)
O1—C12—C13	124.3 (4)	C34—C33—H33	119.6
O1—C12—C17	115.9 (4)	C32—C33—H33	119.6
C13—C12—C17	119.7 (4)	C35—C34—C33	121.0 (4)
C12—C13—C14	119.8 (4)	C35—C34—C39	119.4 (4)
C12—C13—H13	120.1	C33—C34—C39	119.7 (4)
C14—C13—H13	120.1	C36—C35—C34	118.8 (4)
C13—C14—C15	121.8 (4)	C36—C35—H35	120.6
C13—C14—H14	119.1	C34—C35—H35	120.6
C15—C14—H14	119.1	C35—C36—C37	121.0 (4)
C14—C15—C16	116.8 (4)	C35—C36—H36	119.5
C14—C15—C1	124.1 (3)	C37—C36—H36	119.5
C16—C15—C1	119.1 (3)	C36—C37—C32	120.7 (4)
C17—C16—C15	121.3 (4)	C36—C37—H37	119.7
C17—C16—H16	119.3	C32—C37—H37	119.7
C15—C16—H16	119.3	F2—C38—F1	112.1 (8)
C16—C17—C12	120.3 (4)	F2—C38—F3	101.7 (7)
C16—C17—H17	119.8	F1—C38—F3	100.5 (8)
C12—C17—H17	119.8	F9—C38—C28	119.8 (10)
O2—C18—C23	124.9 (4)	F2—C38—C28	116.6 (7)
O2—C18—C19	115.2 (4)	F1—C38—C28	112.6 (6)
C23—C18—C19	119.9 (4)	F3—C38—C28	111.6 (7)
C20—C19—C18	119.9 (4)	C28—C38—F7	107.0 (10)
C20—C19—H19	120.1	F6—C39—F5	110.7 (7)
C18—C19—H19	120.1	F6—C39—F4	109.0 (8)
C19—C20—C21	121.3 (4)	F5—C39—F4	98.3 (7)
C19—C20—H20	119.3	F11—C39—C34	115.1 (7)
C21—C20—H20	119.3	F6—C39—C34	115.4 (5)
C22—C21—C20	118.2 (4)	F5—C39—C34	111.3 (6)
C22—C21—C1	122.9 (4)	F10—C39—C34	108.1 (9)
C20—C21—C1	118.8 (3)	F4—C39—C34	110.9 (5)
C21—C22—C23	121.3 (4)	C6—N1—C5	115.9 (3)
C21—C22—H22	119.4	C7—N2—C8	115.7 (3)
C23—C22—H22	119.4	C12—O1—C24	116.8 (3)
C18—C23—C22	119.4 (4)	C18—O2—C25	117.4 (4)
			
C15—C1—C2—C3	−56.1 (6)	C11—C1—C21—C20	168.0 (3)
C11—C1—C2—C3	−176.8 (4)	C2—C1—C21—C20	−82.8 (4)
C21—C1—C2—C3	65.9 (5)	C20—C21—C22—C23	0.6 (6)
C15—C1—C2—C6	127.9 (4)	C1—C21—C22—C23	−176.0 (4)
C11—C1—C2—C6	7.2 (4)	O2—C18—C23—C22	177.3 (4)
C21—C1—C2—C6	−110.1 (4)	C19—C18—C23—C22	−1.3 (6)
C6—C2—C3—C4	−1.7 (6)	C21—C22—C23—C18	0.4 (6)
C1—C2—C3—C4	−177.5 (4)	N1—C5—C26—C31	59.9 (5)
C2—C3—C4—C5	2.8 (6)	C4—C5—C26—C31	−120.1 (5)
C3—C4—C5—N1	−2.0 (6)	N1—C5—C26—C27	−120.8 (4)
C3—C4—C5—C26	178.0 (4)	C4—C5—C26—C27	59.2 (6)
C3—C2—C6—N1	−0.3 (6)	C31—C26—C27—C28	−1.4 (6)
C1—C2—C6—N1	176.3 (4)	C5—C26—C27—C28	179.3 (4)
C3—C2—C6—C7	178.5 (3)	C26—C27—C28—C29	2.0 (7)
C1—C2—C6—C7	−4.9 (4)	C26—C27—C28—C38	−176.5 (5)
N1—C6—C7—N2	−0.7 (6)	C27—C28—C29—C30	−1.3 (7)
C2—C6—C7—N2	−179.5 (4)	C38—C28—C29—C30	177.2 (5)
N1—C6—C7—C11	178.8 (4)	C28—C29—C30—C31	0.0 (8)
C2—C6—C7—C11	0.0 (4)	C27—C26—C31—C30	0.1 (6)
N2—C8—C9—C10	2.4 (6)	C5—C26—C31—C30	179.4 (4)
C32—C8—C9—C10	−175.5 (4)	C29—C30—C31—C26	0.6 (7)
C8—C9—C10—C11	0.3 (6)	N2—C8—C32—C37	165.7 (4)
N2—C7—C11—C10	3.3 (6)	C9—C8—C32—C37	−16.2 (6)
C6—C7—C11—C10	−176.2 (3)	N2—C8—C32—C33	−15.8 (6)
N2—C7—C11—C1	−175.6 (3)	C9—C8—C32—C33	162.3 (4)
C6—C7—C11—C1	4.9 (4)	C37—C32—C33—C34	1.0 (6)
C9—C10—C11—C7	−3.0 (6)	C8—C32—C33—C34	−177.6 (4)
C9—C10—C11—C1	175.7 (4)	C32—C33—C34—C35	−0.1 (7)
C15—C1—C11—C7	−130.0 (3)	C32—C33—C34—C39	179.4 (4)
C2—C1—C11—C7	−7.3 (4)	C33—C34—C35—C36	−1.0 (7)
C21—C1—C11—C7	106.6 (4)	C39—C34—C35—C36	179.4 (5)
C15—C1—C11—C10	51.2 (5)	C34—C35—C36—C37	1.3 (8)
C2—C1—C11—C10	174.0 (4)	C35—C36—C37—C32	−0.4 (8)
C21—C1—C11—C10	−72.1 (5)	C33—C32—C37—C36	−0.7 (7)
O1—C12—C13—C14	−179.1 (4)	C8—C32—C37—C36	177.8 (4)
C17—C12—C13—C14	2.6 (6)	C29—C28—C38—F2	18.5 (10)
C12—C13—C14—C15	0.0 (6)	C27—C28—C38—F2	−163.0 (8)
C13—C14—C15—C16	−1.2 (6)	C29—C28—C38—F1	150.2 (9)
C13—C14—C15—C1	176.3 (4)	C27—C28—C38—F1	−31.3 (10)
C11—C1—C15—C14	107.7 (4)	C29—C28—C38—F3	−97.7 (8)
C2—C1—C15—C14	−6.1 (6)	C27—C28—C38—F3	80.8 (8)
C21—C1—C15—C14	−127.2 (4)	C35—C34—C39—F6	91.0 (10)
C11—C1—C15—C16	−74.9 (5)	C33—C34—C39—F6	−88.6 (10)
C2—C1—C15—C16	171.3 (4)	C35—C34—C39—F5	−36.2 (7)
C21—C1—C15—C16	50.2 (5)	C33—C34—C39—F5	144.2 (6)
C14—C15—C16—C17	0.0 (6)	C35—C34—C39—F4	−144.6 (8)
C1—C15—C16—C17	−177.7 (4)	C33—C34—C39—F4	35.9 (10)
C15—C16—C17—C12	2.6 (7)	C2—C6—N1—C5	1.1 (6)
O1—C12—C17—C16	177.7 (4)	C7—C6—N1—C5	−177.5 (3)
C13—C12—C17—C16	−3.8 (7)	C4—C5—N1—C6	0.0 (5)
O2—C18—C19—C20	−177.6 (4)	C26—C5—N1—C6	−180.0 (3)
C23—C18—C19—C20	1.2 (6)	C11—C7—N2—C8	−0.7 (6)
C18—C19—C20—C21	−0.1 (6)	C6—C7—N2—C8	178.8 (3)
C19—C20—C21—C22	−0.8 (6)	C9—C8—N2—C7	−2.2 (6)
C19—C20—C21—C1	176.0 (4)	C32—C8—N2—C7	175.8 (3)
C15—C1—C21—C22	−140.5 (4)	C13—C12—O1—C24	24.3 (6)
C11—C1—C21—C22	−15.4 (5)	C17—C12—O1—C24	−157.3 (4)
C2—C1—C21—C22	93.8 (4)	C23—C18—O2—C25	5.1 (6)
C15—C1—C21—C20	42.9 (5)	C19—C18—O2—C25	−176.2 (4)

Symmetry codes: (i) −*x*+1/2, −*y*, *z*+1/2; (ii) −*x*−1/2, −*y*, *z*+1/2.
